# A Longitudinal Study on Generalized Anxiety Among University Students During the First Wave of the COVID-19 Pandemic in Switzerland

**DOI:** 10.3389/fpsyg.2021.643171

**Published:** 2021-03-11

**Authors:** Simone Amendola, Agnes von Wyl, Thomas Volken, Annina Zysset, Marion Huber, Julia Dratva

**Affiliations:** ^1^School of Applied Psychology, Zurich University of Applied Sciences, Zurich, Switzerland; ^2^Department of Dynamic and Clinical Psychology, Faculty of Medicine and Psychology, Sapienza University of Rome, Rome, Italy; ^3^School of Health Professions, Zurich University of Applied Sciences, Winterthur, Switzerland; ^4^Medical Faculty, University of Basel, Basel, Switzerland

**Keywords:** anxiety, longitudinal, lockdown, coronavirus, COVID-19 pandemic, linear mixed model

## Abstract

**Objective:**

The COVID-19 pandemic and government measures implemented to counter the spread of the infection may be a major stressor affecting the psychological health of university students. This study aimed to explore how anxiety symptoms changed during the pandemic.

**Methods:**

676 students (76% females) at Zurich University of Applied Sciences participated in the first (T0) and second (T1) survey waves. Anxiety symptoms were assessed using the Generalized Anxiety Disorder-Scale-7 (GAD-7). Risk and protective factors (e.g., COVID-19-related variables) were examined.

**Results:**

GAD-7 scores decreased significantly from T0 to T1 (mean change: −0.446, SE = 0.132, 95% CI: −0.706, −0.186, *t* = −3.371, *df* = 659, *p* = 0.001). Participants with moderate-to-severe anxiety score were 20.2 and 15.6% at T0 and T1, respectively. The following positively predicted anxiety: older age, female gender, non-Swiss nationality, loneliness, participants’ concern about their own health, and interaction between time and participants’ concern about their own health. Resilience and social support negatively predicted anxiety.

**Conclusions:**

Our findings provide information for public health measures and psychological interventions supporting the mental health of university students during the COVID-19 emergency.

## Introduction

**FIGURE 1:**
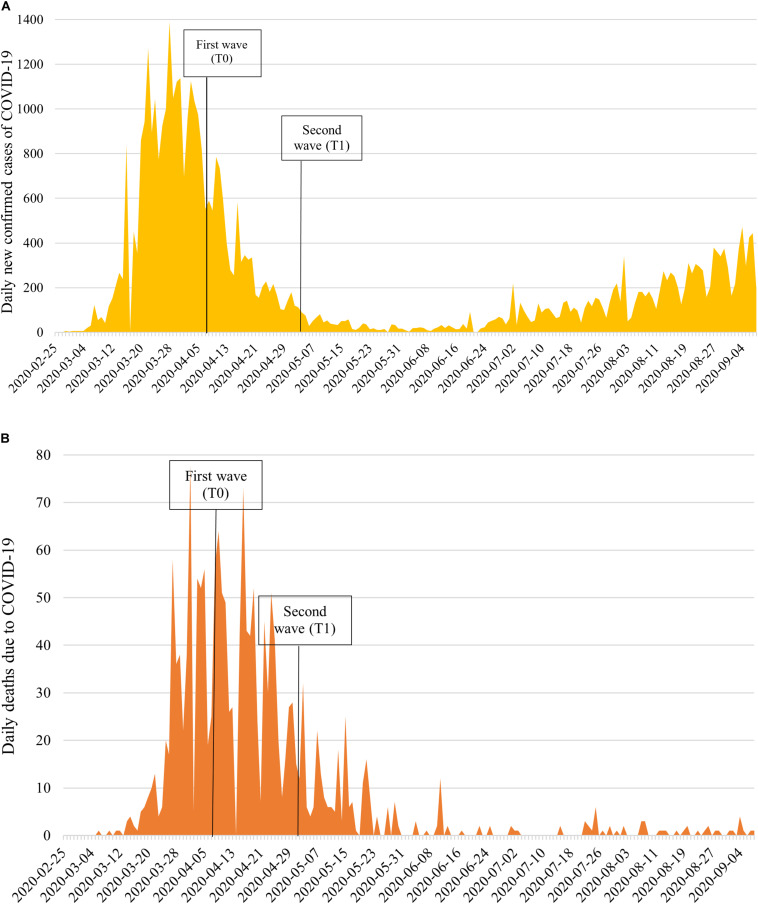
National epidemic trend of the daily number of: **(A)** People testing positive for SARS-Cov-2, and **(B)** Deaths due to COVID-19 in Switzerland from February 25–September 7, 2020.

On March 13, 2020, to contain the spread of severe acute respiratory syndrome coronavirus 2 (SARS-CoV-2), which causes coronavirus disease 2019 (COVID-19), and protect the population, the Swiss government (Federal Council, 2020) canceled face-to-face educational activities, banned all events involving more than 100 people, decided to partially close its borders, and implemented border controls. The government subsequently banned non-essential retail commercial activities and gatherings of more than 5 people. Based on health monitoring of the spread of COVID-19 within Switzerland, 1,103,149 tests for COVID-19 confirmed 44,592 cases from the beginning of the outbreak up to September 7, 2020. Figure 1 shows the trend of the COVID-19 pandemic in Switzerland from February 25 to September 7, 2020. On April 3, there were 20,505 confirmed cases and 666 deaths due to COVID-19 in Switzerland. The cumulative number of cases and deaths over the previous week (i.e., from March 28 to April 3) were 6,429 and 366, respectively. On April 30, confirmed COVID-19 cases and deaths were 29,705 and 1,754, respectively. Confirmed COVID-19 cases and deaths over the previous week were 811 and 155, respectively (i.e., from April 23 to April 30).

Sudden or unexpected stressful and potentially dangerous natural events can cause an increase in mental distress in adults (McLaughlin et al., 2010). Previous studies showed increased levels of psychological distress (e.g., anxiety, anger) during isolation for Middle East Respiratory Syndrome (MERS) (Jeong et al., 2016) and during the outbreak of novel swine-origin influenza A (H1N1) (Jones and Salathé, 2009). Moreover, anxiety levels closely mirrored the daily number of new cases during the 2003 outbreak of severe acute respiratory syndrome (SARS) in Hong Kong (Leung et al., 2005).

The adaptive function of the normal anxiety response is to prepare the individual to detect and cope with threats or danger (Bateson et al., 2011). Genetic, psychosocial, and environmental factors influence the propensity and persistence of the anxiety response and the emergence of clinical anxiety (Hettema et al., 2005; Bergstrom and Meacham, 2016; Fullana et al., 2020; Zimmermann et al., 2020b). Stressful events represent an important risk factors for the emergence of anxiety symptoms and difficulties in regulating negative emotions (Anyan et al., 2017; Ding et al., 2020; Schneider et al., 2020). Furthermore, feelings of loneliness, intolerance of uncertainty, worry and fear generalization are related to symptoms of anxiety (Dar et al., 2017; Hamm, 2019; Lauriola et al., 2019; Stegmann et al., 2019; Danneel et al., 2020). Recent studies showed that 29 and 24% of the general population reported moderate-to-severe symptoms of anxiety during the initial outbreak of COVID-19 in China (Wang et al., 2020) and the United Kingdom (Fancourt et al., 2020), respectively. The percentages of moderate-to-severe anxiety in those countries were lower before the COVID-19 pandemic (Huang et al., 2019; Giebel et al., 2020). Importantly, recent findings raise concern for the mental health of university students. In Jordan, Naser et al. (2020) looked at depression and anxiety and observed a higher prevalence of anxiety among university students (38 and 21%, respectively) than among healthcare professionals (21 and 11%) and among university students than in the general population (16 and 9%). Naser et al. see a possible explanation for these results in students’ major concerns about the impact of the pandemic on their university education and performance. An increase in symptoms of anxiety has also been registered among Chinese and Greek university students (Kaparounaki et al., 2020; Wang and Zhao, 2020).

Although the acute impact of the pandemic on students’ psychological well-being has been confirmed in the literature, most of the available studies are cross-sectional. To date, few longitudinal studies have explored symptoms of anxiety among university students during the COVID-19 pandemic. Findings on changes in anxiety levels are mixed. One study (Li et al., 2020) observed a decrease in symptoms of anxiety and depression after 2 weeks of confinement measures to minimize spread of the coronavirus, whereas two studies (Elmer et al., 2020; Zimmermann et al., 2020a) indicated an increase in the severity of anxiety.

In light of the above, the first aim of the present study was to increase knowledge on the course of symptoms of anxiety in Swiss university students during the COVID-19 outbreak. This issue deserves more attention, as previous studies found close relationships between psychological distress, poor academic performance, and career outcomes (Tartas et al., 2011; Raskind et al., 2019). Moreover, symptoms of anxiety can lead to later adverse mental health outcomes and reduced quality of life (Fichter et al., 2010; Kasteenpohja et al., 2018). We hypothesized that anxiety symptoms in Swiss university students were higher when daily COVID-19 cases and deaths were constantly increasing (i.e., at T0) than when they were decreasing (i.e., at T1). The second aim of our study was to explore a wide range of individual and contextual factors to identify risk and protective factors in anxiety during the pandemic.

## Materials and Methods

### Participants and Procedure

Students at the Zurich University of Applied Sciences (ZHAW) (*N* = 13,500) in Switzerland were invited to participate in a web survey exploring the impact of the COVID-19 pandemic on students’ psychophysical health. In the present study, we report the results of the analysis of responses concerning impact on mental health by university students who participated at both the first (T0) and second (T1) wave (*N* = 676). The surveys lasted about 20–25 min and ran for a total of 7 working days (i.e., from April 3–14, 2020 for the first wave; from April 30 to May 11, 2020 for the second wave).

Methods and anxiety symptoms at baseline have been published in detail in Dratva et al. (2020). The study sample involved students (*N* = 2,429) from all ZHAW faculties despite students from the school of health professions and social work were slightly overrepresented (35 and 31% of the total sample at baseline, respectively) (Dratva et al., 2020). Furthermore, a total of 70% were female students and the median age was 25 years (interquartile range 23–28) (Dratva et al., 2020).

Participants’ informed consent was obtained before starting the survey. Anonymity of participants was ensured by asking them to generate a personal code at the start of the web survey for the merging of follow-up survey data. The study was approved by both the local cantonal ethics committee (BASEC-Nr. Req-2020-00326) and the ZHAW data protection officer.

### Measures

*Sociodemographic and COVID-19-related variables*. Participants provided sociodemographic information at T0, including age, gender, degree program (i.e., BSc or MSc), social status of parents at student age 16 years, and nationality.

A set of questions on COVID-19 related concerns and students’ life was partly specifically developed for this target group and their context and partly adapted from previous studies (Essadek and Rabeyron, 2020; Sotomo, 2020; Wathelet et al., 2020). The questions specifically designed for this study was developed by the authors representing researchers, lecturers and students. Five students of different faculties piloted the questionnaire reporting any technical and content issues.

Students were asked at T0 and T1 about the effects of the COVID-19 pandemic and the public health measures on their student and everyday life. They were asked to agree or not agree with statements by responding on a 5-point Likert scale ranging from 1 (*completely disagree*) to 5 (*completely agree*). Students’ worry about semester completion and a feeling of loneliness in everyday life were explored (i.e., “I am worried about my semester completion” and “I am lonely,” respectively). Responses were then dichotomized as 0 (i.e., *I disagree completely, I tend to disagree, neutral or partially disagree and partially agree, not relevant*) and 1 (i.e., *I tend to agree, I completely agree*).

We assessed the concerns that students had about themselves or their family (parents, siblings, grandparents, their own child/child of partner, other relatives) in the context of COVID-19 at T0 and T1. Response options for the question, “Are you concerned about your own health in the context of the pandemic?” (and “Are you concerned about your [family member] health in the context of the pandemic?”) were 1 (*I have no concerns*), 2 (*some concerns*), 3 (*big concerns*), and not relevant. A question about concerns about their family members’ (omitting their child/child of partner) financial situation was also presented in the same manner. Responses were dichotomized as 0 (*I have no concern, I have some concerns, not relevant*) and 1 (*I have big concerns*) for concern for their own health, for significant others’ health, and for family members’ financial situation.

Finally, students’ symptoms and testing for COVID-19 were assessed using the following statements with dichotomous response options (*no, yes*) at T0 and T1: “Have you had symptoms in the past 4 weeks that would be compatible with a COVID-19 infection? For example, cough (usually dry), sore throat, shortness of breath, and fever, muscle pain”; “Have you had a COVID-19 test in the past 4 weeks?”; “Have you tested positive for COVID-19?”.

*Alcohol and marijuana consumption* (Hibell et al., 2009). Participants were asked about binge drinking behavior at T0, i.e., how many times (if any) they had drunk 5 or more units of alcohol on one occasion during the past 30 days [i.e., one unit is a glass of beer (about 0.5 L) or a glass of wine/sparkling wine (about 0.2 L) or a bottle of alcopop (about 0.33 L) or a glass of spirits (about 0.04 L)]. Participants responded on a 6-point Likert scale ranging from 1 (*never*) to 6 (*10 or more times*). The answer was dichotomized as 0 (*never*) and 1 (*at least once*).

Marijuana consumption in the past 30 days was also explored at T0 (adapted from Hibell et al., 2009). Participants responded on a 9-point Likert scale ranging from 1 (*I do not use it*) to 9 (*10 or more times*). The answer was dichotomized as 0 (*no use*) and 1 (*at least once*).

The *Oslo Social Support Scale* (OSSS-3) (Dalgard, 1996) is a short questionnaire to explore social support through three items on the number of close confidants, sense of concern or interest from other people, and relationship to neighbors (Kocalevent et al., 2018). High values represent strong levels of social support. In the present study, Cronbach’s alpha was 0.53.

The *Brief Resilient Coping Scale* (BRCS) (Sinclair and Wallston, 2004) is a brief self-report questionnaire that assesses resilient coping conceptualized as the tendency to cope with stress in a highly adaptive manner. It comprises four items. Participants respond on a 5-point Likert scale (from 1 (*does not describes me at all*) to 5 (*describes me very well*). The total score varies between 4 to 20, with higher scores indicating higher resilience. In the present study, Cronbach’s alpha was 0.59.

The *Generalized Anxiety Disorder-Scale-7* (GAD-7) (Spitzer et al., 2006) is a self-report questionnaire that explores the anxiety level as experienced by participants in the last 2 weeks. Anxiety symptoms were investigated at T0 and T1. The GAD-7 includes seven items to be rated on a 4-point Likert scale 0 (*not at all*) to 3 (*nearly every day*). The total score ranges from 0 to 21, with higher scores indicating higher levels of anxiety. Furthermore, the resulting score could be categorized into four levels of anxiety: minimal (0–4), mild (5–9), moderate (10–14) and severe (15–21). In the present study, Cronbach’s alpha was 0.86 at T0 and 0.88 at T1.

### Statistical Analyses

Descriptive statistics (i.e., frequencies, prevalence, mean, standard deviation) were applied to evaluate the characteristics of the sample. Univariate analysis of variance (ANOVA) and Chi-square test of independence were used to investigate differences according to gender. Cramer’s V was used to express effect size in the latter analyses.

Mean-level stability was analyzed with paired samples T-tests and differential stability with Pearson correlations between baseline (T0) and follow-up (T1) anxiety scores. We used linear mixed models (LMMs) to examine changes in anxiety symptoms over time and the associations between anxiety and a set of time-constant covariates (i.e., predictors measured at baseline T0: age, gender, degree program, social status of parents, nationality, social support, resilient coping, binge drinking, marijuana use) as well as a set of time-varying covariates (i.e., measured at T0 and T1: worry about completing the semester, feeling of loneliness, concern for their own health, concern for family members’ health, and concern for family members’ financial situation, COVID-19 symptoms). The continuous outcome model included a random (subject−specific) intercept and an autoregressive model of order 1 for the residuals within participants using maximum likelihood estimates of parameters. All continuous predictor variables were mean-centered before they were entered into the LMMs.

Initially, all predictors of anxiety symptoms were fitted separately; in the final model, all measures were fitted jointly to determine the unique relevance of predictors after accounting for the influence of all other predictors. Estimated marginal mean scores and standard error were reported examining the significant effect of interaction terms.

All data were analyzed using SPSS Version 25. *P* values <0.05 were considered statistically significant.

## Results

Table 1 reports the sociodemographic, COVID-19-related, and psychosocial characteristics of the study population; 76% (*n* = 514) of the sample participants were women.

**TABLE 1:** Sample’s characteristics at the first (T0) and second wave (T1).

	T0			T1		
Variable	Total *n* (%)	Men *n* (%)	Women *n* (%)	Total *n* (%)	Men *n* (%)	Women *n* (%)
Sociodemographic						
Age (M ± SD)	26.67 (5.83)	26.58 (4.29)	26.71 (6.24)	–	–	–
Pursued degree (Master’s)	94 (13.9)	15 (9.3)	79 (15.4)	–	–	–
Parents’ social status (M ± SD)	5.66 (1.55)	5.74 (1.48)	5.64 (1.57)	–	–	–
Nationality (Switzerland)	602 (93.9)	141 (95.9)	460 (93.3)	–	–	–
COVID-19-related						
Worry about completing semester^a^	308 (45.6)	69 (42.9)	238 (46.3)	415 (61.4)	86 (53.4)	328 (63.8)
Feeling lonely^b^	203 (30.0)	64 (40.0)	139 (27.6)	201 (29.7)	52 (32.7)	149 (29.2)
Health concern	16 (2.4)	1 (0.6)	15 (2.9)	15 (2.2)	2 (1.2)	13 (2.5)
Health concern for others ^a,b^	300 (44.4)	51 (31.7)	249 (48.4)	251 (37.1)	40 (24.8)	211 (41.1)
Financial concern for others	125 (18.5)	22 (13.7)	103 (20.0)	94 (13.9)	17 (10.6)	77 (15.0)
Binge drinking^b^	146 (21.6)	55 (43.0)	91 (22.9)	–	–	–
Marijuana use	65 (9.6)	19 (12.8)	45 (9.0)	–	–	–
COVID-19 symptoms	128 (18.9)	26 (17.6)	102 (20.4)	60 (8.9)	9 (5.6)	51 (9.9)
COVID-19 test	13 (1.9)	3 (2)	9 (1.8)	11 (1.6)	0 (0)	11 (2.1)
COVID-19 positive test	2 (0.3)	0 (0)	2 (1.35)	1 (0.1)	–	1 (0.19)
Mental health						
OSSS-3 (M ± SD)	10.48 (1.82)	9.93 (1.79)	10.65 (1.80)	–	–	–
BRCS (M ± SD)	15.38 (2.24)	15.41 (2.02)	15.36 (2.30)	–	–	–
GAD-7 (M ± SD)	6.30 (4.30)	5.44 (4.07)	6.57 (4.35)	5.87 (4.39)	5.25 (4.08)	6.05 (4.47)

**OSS-3*, Oslo Social Support Scale; *BRCS*, Brief Resilient Coping Scale; *GAD-7*, Generalized Anxiety Disorder Scale. ^*a*^Significant gender difference (*p* < 0.05) at T1. ^*b*^Significant gender difference (*p* < 0.05) at T0.*

### COVID-19-Related Factors

At T0, 18.9% (*n* = 128) of participants had symptoms in the past 4 weeks compatible with COVID-19 infection, 1.9% (*n* = 13) had a COVID-19 test, and two participants tested positive for the disease. At T1, 8.9% (*n* = 60) of participants had symptoms in the past 4 weeks compatible with COVID-19 infection, 1.6% (*n* = 11) had a COVID-19 test, and one participant tested positive for the disease.

Regarding the effect of the COVID-19 pandemic on the students’ academic life, almost half (45.6%, *n* = 308) of the sample felt worried about completing the semester at T0 and more than half (61.4%, *n* = 415) at T1. A significant gender difference was detected only at T1 (χ^2^ = 5.59, *df* = 1, *p* = 0.018, Cramer’s V = 0.091). More women (63.8%, standardized residual = 0.7) reported worry about completing the semester than men (53.4%, standardized residual = −1.3). Furthermore, about a third (T0: 30%, *n* = 203; T1: 29.7%, *n* = 201) reported feeling lonely in everyday life, with gender difference only at T0 (χ^2^ = 8.83, *df* = 1, *p* = 0.003, Cramer’s *V* = 0.115). More men (40.0%, standardized residual = 2.2) than women (27.6%, standardized residual = −1.2) experienced loneliness at baseline.

A small percentage (T0: 2.4%, *n* = 16; T1: 2.2%, *n* = 15) of the study population showed high concern for their own health. 44.4% (*n* = 300) and 37.1% (*n* = 251) of the students reported to be worried about the health of family members at T0 and T1, respectively. Health concern for family members was associated with gender at both T0 (χ^2^ = 13.96, *df* = 1, *p* < 0.001, Cramer’s *V* = 0.144) and T1 (χ^2^ = 13.78, *df* = 1, *p* < 0.001, Cramer’s *V* = 0.143). More women (T0: 48.4%, standardized residual = 1.4; T1: 41.1%, standardized residual = 1.4) than men (T0: 31.7%, standardized residual = −2.4; T1: 24.8%, standardized residual = −2.6) were worried about the health of family members.

Furthermore, 18.5% (*n* = 125) and 13.9% (*n* = 94) of the students reported being worried about the financial situation of family members at T0 and T1, respectively.

Binge drinking and marijuana consumption (i.e., at least on one occasion) was reported by 21.6 and 9.6% of the students, respectively, during the past month at T0. Only binge drinking was associated with gender (χ^2^ = 19.52, *df* = 1, *p* < 0.001, Cramer’s *V* = 0.193). More men (43%, standardized residual = 3.3) than women (22.9%, standardized residual = −1.9) showed binge drinking behavior.

### Symptoms of Anxiety: Risk and Protective Factors

Table 2 shows the prevalence of anxiety levels according to cut-off scores provided by the authors of the GAD-7. Participants with moderate-to-severe anxiety score decreased from 20.2% (*n* = 133) to 15.6% (*n* = 104) over the 1-month period.

**TABLE 2:** Prevalence of anxiety levels at the first (T0) and second wave (T1).

	T0			T1		
Anxiety level	Total *n* (%)	Men *n* (%)	Women *n* (%)	Total *n* (%)	Men *n* (%)	Women *n* (%)
Minimal	264 (40.2)	75 (49.0)	189 (37.5)	309 (46.1)	84 (52.8)	225 (44.1)
Mild	260 (39.6)	54 (35.3)	206 (40.9)	257 (38.4)	58 (36.5)	199 (39.0)
Moderate	98 (14.9)	18 (11.8)	80 (15.9)	60 (9.0)	9 (5.7)	50 (9.8)
Severe	35 (5.3)	6 (3.9)	29 (5.8)	44 (6.6)	8 (5.0)	36 (7.1)

*18 and 6 participants missed the first and second wave, respectively.*

Mean-level stability of anxiety scores across the first (T0) and second wave (T1) was very high (Cohen’s *d* = 0.10), and differential stability was high (*r* = 0.697, *p* < 0.001).

**FIGURE 2:**
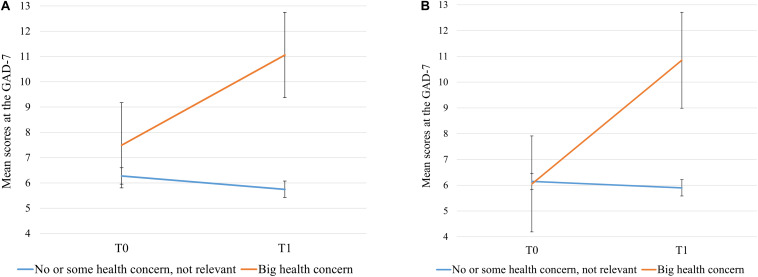
Estimated marginal mean scores at the Generalized Anxiety Scale - 7 as predicted by the interaction term Time * participant’s Concern for their own health in: **(A)** Unadjusted Model 1, and **(B)** Adjusted Model 2. Error bars represent the 95% confidence intervals.

Nearly all factors were found to individually predict symptoms of anxiety when fitted separately, except for pursued degree, binge drinking and marijuana use during the past 30 days, and having had symptoms compatible with COVID-19 infection (Table 3, Model 1). A significant effect of time was found for anxiety symptom severity. GAD-7 scores decreased significantly from T0 to T1 (mean change: −0.446, SE = 0.132, 95% CI of the difference: −0.706, −0.186, *t* = −3.371, *df* = 659, *p* = 0.001). Furthermore, the interaction effect of Time ^∗^ participants’ Health concern for their own health was statistically significant. Specifically, Figure 2A shows that symptoms of anxiety were stable or decreased slightly in participants with no or some concern about their own health over the 1-month period (M = 6.27, SE = 0.17 at T0; M = 5.75, SE = 0.17 at T1), whereas symptoms of participants who reported high health concern increased (M = 7.49, SE = 0.81 at T0; M = 11.06, SE = 0.86 at T1).

**TABLE 3:** Estimated fixed effects of individual and contextual predictors of anxiety symptoms.

Variable	Model 1: all fitted separately			Model 2: all fitted jointly		
	β (SE)	95% CI	*p*-value	β (SE)	95% CI	*p*-value
Time	−0.446 (0.132)	−0.706, −0.186	0.001	−0.987 (0.635)	−2.236, 0.262	0.121
Sociodemographic						
Age	0.064 (0.026)	0.012, 0.115	0.016	0.076 (0.025)	0.027, 0.125	0.003
Gender (male)	−0.972 (0.361)	−1.681, −0.264	0.007	−1.048 (0.345)	−1.726, −0.369	0.003
Pursued degree (Master’s)	−0.217 (0.445)	−1.090, 0.656	0.626	−0.635 (0.425)	−1.470, 0.200	0.136
Parents’ social status	−0.348 (0.101)	−0.548, −0.150	0.001	−0.186 (0.097)	−0.375, 0.004	0.055
Nationality (other than Swiss)	1.616 (0.654)	0.331, 2.900	0.014	1.531 (0.605)	0.343, 2.719	0.012
Psychosocial						
Social support	−0.468 (0.085)	−0.635, −0.301	<0.001	−0.299 (0.083)	−0.463, −0.136	<0.001
Resilient coping	−0.574 (0.067)	−0.705, −0.444	<0.001	−0.373 (0.066)	−0.503, −0.242	<0.001
COVID-19-related						
Worry about completing semester	−0.443 (0.196)	−0.826, −0.059	0.024	−0.374 (0.539)	−1.433, 0.685	0.488
Feeling lonely	1.967 (0.229)	1.517, 2.416	<0.001	1.658 (0.576)	0.527, 2.788	0.004
Health concern	3.170 (0.674)	1.847, 4.493	<0.001	−5.139 (1.996)	−9.058, −1.221	0.010
Health concern for others	1.199 (0.212)	0.782, 1.615	<0.001	0.881 (0.542)	−0.183, 1.944	0.104
Financial concern for others	1.734 (0.303)	1.139, 2.329	<0.001	0.781 (0.674)	−0.543, 2.105	0.247
Binge drinking	0.289 (0.381)	−0.460, 1.038	0.449	0.589 (0.329)	−0.057, 1.236	0.074
Marijuana use	0.661 (0.526)	−0.372, 1.694	0.209	0.352 (0.469)	−0.569, 1.274	0.453
COVID-19 symptoms	0.185 (0.286)	−0.377, 0.747	0.518	−0.416 (0.712)	−1.815, 0.983	0.559
Interactions						
Time * Worry about completing semester	0.272 (0.314)	−0.343, 0.888	0.385	0.251 (0.336)	−0.409, 0.910	0.456
Time * Feeling lonely	0.126 (0.330)	−0.522, 0.773	0.703	0.145 (0.351)	−0.544, 0.833	0.680
Time * Health concern	4.095 (1.015)	2.103, 6.087	<0.001	5.044 (1.267)	2.557, 7.532	<0.001
Time * Health concern for others	0.058 (0.303)	−0.537, 0.652	0.849	−0.056 (0.336)	−0.715, 0.603	0.868
Time * Financial concern for others	−0.124 (0.394)	−0.899, 0.650	0.754	0.335 (0.428)	−0.506, 1.176	0.435
Time * COVID-19 symptoms	0.176 (0.466)	−0.738, 1.091	0.705	0.459 (0.505)	−0.534, 1.452	0.364

*SE, standard error; CI, confidence interval.*

When all factors were then jointly modeled (Table 3, Model 2), the effect of time, social status of parents, worry about completing the semester, and participants’ health and financial concerns for family members on anxiety symptoms became statistically non-significant. However, social status (*p* = 0.055) and binge drinking (*p* = 0.074) were borderline significant (Bland, 2015), indicating that a higher social status of parents at student age 16 years was associated with lower anxiety scores and that binge drinking was associated with higher anxiety scores. An older age, female gender, nationality other than Swiss, feeling of loneliness, and participants’ concern for their own health significantly predicted higher symptoms of anxiety. On the other hand, resilient coping and social support were protective factors for symptoms of anxiety. Finally, the interaction effect of Time ^∗^ participants’ Health concern for their own health remained statistically significant. Namely, symptoms of anxiety were stable in participants with no or some concern for their own health over the 1-month period (M = 6.15, SE = 0.16 at T0; M = 5.90, SE = 0.16 at T1) and increased in participants with high health concern (M = 6.05, SE = 0.95 at T0; M = 11.85, SE = 1.01 at T1) (Figure 2B).

## Discussion

Our study, to our knowledge one of few longitudinal studies on anxiety in university students during the COVID-19 pandemic, indicates a decrease in anxiety symptoms with time and decreasing population infection rates. However, we see heterogenous trends in individuals’ concern for their own heath in the pandemic.

In the first wave, considering the GAD-7 cut-off, 20.2% of students reported moderate-to-severe anxiety. This result is in line with previous studies (Chi et al., 2020; Naser et al., 2020; Perz et al., 2020; Savitsky et al., 2020; Wang and Zhao, 2020; Zhang et al., 2020) that found high levels of symptoms of anxiety among university students during the COVID-19 pandemic. The prevalence of moderate-to-severe anxiety among our sample of participants decreased to 15.6% at the second wave (i.e., after 1 month).

The findings of this present study reveal a significant effect of time on anxiety symptom severity. Anxiety symptoms decreased 0.45 points according to the GAD-7 mean scores between the first and second wave, which supports our first hypothesis. This finding is consistent with that of a study conducted in China (Li et al., 2020). However, two studies observed an increase in symptoms of anxiety (Elmer et al., 2020; Zimmermann et al., 2020a). The differences in the studies’ results could be explained by the timing of the data collection periods and the respective trends of the COVID-19 infection. In particular, Elmer et al. (2020) observed an increase of anxiety during the COVID-19 emergency (i.e., April 2020) compared to pre-emergency levels (i.e., April and September 2019). Zimmermann et al. (2020a) found an increase in anxiety symptoms simultaneously with a consistent daily increase in the number of new infections and deaths from COVID-19 in the United States. In contrast, Li et al. (2020) as well as the present study found a decrease in anxiety symptoms that corresponds with a decline in the number of the newly infected and the number of deaths. These results are in line with the prediction that if the probability of incurring threats to survival increases (i.e., spread of the COVID-19 infection), the severity of anxiety symptoms increases (Bateson et al., 2011).

Importantly, mean GAD-7 scores (at both T0 and T1) among our sample of participants as well as in another Swiss sample (Elmer et al., 2020) were lower than those observed in other two studies using the same measure of anxiety among university and college students during the COVID-19 pandemic (Liu et al., 2020; Perz et al., 2020; Savitsky et al., 2020; Zimmermann et al., 2020a). Biological, psychological, and cultural factors influence the phenomenological presentation and clinical severity of anxiety (Kirmayer et al., 1995; Hettema et al., 2005; Heinrichs et al., 2006; Bergstrom and Meacham, 2016; Fullana et al., 2020; Zimmermann et al., 2020b). The difference in GAD-7 mean scores outlined here may also be related to the country-specific quarantine measures. Cross-cultural studies are needed to further examine this important issue, with its implications for public health measures and health.

From a broader mental health perspective, it would be highly informative to compare our findings with those observed among samples of participants of other countries that were similarly affected by the COVID-19 pandemic. This comparison would improve our understanding of the link between the spread and consequences of the infection in the territory and the changes in psychological health of the population. Findings of a recent study showed that Austria, Switzerland and Portugal experienced a similar low effect of the pandemic on overall deaths between January and May 2020 while a medium-to-high effect was shown for France, Netherlands, Sweden (medium effect), Belgium, Italy, Scotland, Spain, England and Wales (high effect) (Kontis et al., 2020). To date, however, longitudinal studies exploring changes in symptoms of anxiety or mental health among students or general populations are lacking both in Austria and Portugal.

Linear mixed model analysis highlighted different personal and contextual factors associated with the severity of anxiety symptoms during the spread of COVID-19 in Switzerland. Regarding sociodemographic factors, in line with the findings of Li et al.’s (2020) study, we found that an older age was positively associated with symptoms of anxiety among Swiss university students. However, in contrast to the results highlighted by Li et al., we did not observe an effect of the type of the university degree program attended (i.e., Bachelor’s or Master’s degree program) on symptoms of anxiety. A possible explanation for our finding is that older university students may be more concerned that the outbreak could delay their academic career and entry into the labor market or negatively affect their own financial situation, regardless the type of the degree pursued. Elmer et al. (2020) found that students’ worries about their own future career positively predicted anxiety symptoms.

Female gender was also associated with a higher risk of anxiety. This result is in line with some previous studies conducted during the COVID-19 pandemic (Elmer et al., 2020; Naser et al., 2020; Wang and Zhao, 2020), although other studies did not find a gender effect on anxiety (Cao et al., 2020; Li et al., 2020; Zimmermann et al., 2020a). In particular, women compared to men are more likely to suffer from anxiety due to differences in risk and protective factors and in clinical presentation (Toufexis et al., 2006; Baxter et al., 2013; Christiansen, 2015).

University students of non-Swiss nationality (e.g., international students, students whose parents do not have Swiss citizenship) were also at increased risk of anxiety during the pandemic. However, Savitsky et al. (2020) did not find evidence for this association. One possible explanation for our finding is that non-Swiss students are not together with their family and other close people who can provide security and support. A second possible reason is related to the concepts of familiarity and “non-territory” (Price, 2003). International students, currently living away from home, may perceive greater insecurity that is linked to the unfamiliarity of the surrounding environment. Finally, some students probably had concerns about relatives living in countries with a high incidence of COVID-19 cases and deaths. Our finding suggests that the impact of the COVID-19 emergency on the mental health of students of non-Swiss nationality (e.g., international students) should be monitored and addressed, and it requires additional research.

In this study, COVID-19-related factors, namely, participants’ concern for the health and financial situation of family members or friends (in the unadjusted model), feeling of loneliness, and concern for their own health (in the final adjusted model), predicted higher symptoms of anxiety among Swiss university students during the pandemic. Moreover, participants who reported high concern for their own health at T0 scored higher on anxiety at T1 compared to participants who were not worried about their own health. These findings are in line with previous studies (McIntyre et al., 2018; Cao et al., 2020; Li et al., 2020; Liu et al., 2020; Naser et al., 2020). Students were more worried about the health of family members than about their own health, as has been previously suggested (Maaravi and Heller, 2020). Further, students’ worry about the health of family members was higher than their concern about family members’ financial situation. Our findings have clinical implications; treatment of students’ anxiety should consider the role of the pandemic-related concern about health and finances in predicting the severity of anxiety symptoms.

Finally, social support and resilient coping negatively predicted university students’ symptoms of anxiety. These findings are consistent with previous studies (Cao et al., 2020; Chi et al., 2020; Liu et al., 2020) and have implications for public health measures during states of emergency. Interventions should be implemented to boost the ability of university students to face stressful situations in an adaptive manner as well as to provide them with social support. It is best to implement the interventions before a crisis occurs, in the sense of preventive measures. As public health and safety measures during pandemics might limit personal meetings with professionals, it is important to ensure that students have the possibility and the infrastructures to take advantage of counseling or psychotherapy interventions.

The findings of this study should be interpreted while keeping some limitations in mind. The use of self-report measures could have increased the risk of social desirability response bias affecting the results. At the same time, the decrease in symptoms of anxiety could be due in part to repeated administration of the self-report measure. Finally, an imbalance between the number of men and women participating in this study was present. Gender imbalance is not uncommon in questionnaire studies. A possible explanation is related to an “interest” bias: students from ZHAW faculties with a high percentage of women (Health Sciences, Psychology and Economics faculties) who were potentially more interested in health-related subjects were more likely to participate (lower response rates from students of Engineering, Life Sciences, Facility Management faculties). Therefore, there are some limitations in the generalizability of the results to the entire ZHAW student population. Further caution must be taken regarding the generalizability of our findings to the broader population of Swiss university students.

Despite these limitations, our study demonstrates the change in symptoms of anxiety and contributes new evidence on the role of individual and contextual factors in predicting anxiety over a 1-month period during the COVID-19 pandemic. These findings can be used to inform both public health measures and psychological treatment supporting psychological well-being of university students during public health emergencies.

## Data Availability Statement

The raw data supporting the conclusions of this article will be made available by the authors, without undue reservation.

## Ethics Statement

The studies involving human participants were reviewed and approved by Local Cantonal Ethics Committee (BASEC-Nr. Req-2020-00326) and ZHAW data protection officer. The patients/participants provided their written informed consent to participate in this study.

## Author Contributions

SA, AW, TV, AZ, MH, and JD contributed to the conceptualization of the study. SA analyzed the data with TV’s support and wrote the original draft of the manuscript. All authors discussed the results and commented on the manuscript approving its final version.

## Conflict of Interest

The authors declare that the research was conducted in the absence of any commercial or financial relationships that could be construed as a potential conflict of interest.

## References

[B1] Anyan F. Worsley L. Hjemdal O. (2017). Anxiety symptoms mediate the relationship between exposure to stressful negative life events and depressive symptoms: a conditional process modelling of the protective effects of resilience. *Asian J. Psychiatr.* 29 41–48. 10.1016/j.ajp.2017.04.019 29061426

[B2] Bateson M. Brilot B. Nettle D. (2011). Anxiety: an evolutionary approach. *Can. J. Psychiatry* 56 707–715. 10.1177/070674371105601202 22152639

[B3] Baxter A. J. Scott K. M. Vos T. Whiteford H. A. (2013). Global prevalence of anxiety disorders: a systematic review and meta-regression. *Psychol. Med.* 43 897–910. 10.1017/S003329171200147X 22781489

[B4] Bergstrom C. T. Meacham F. (2016). Depression and anxiety: maladaptive byproducts of adaptive mechanisms. *Evol. Med. Public Health* 1 214–218. 10.1093/emph/eow019 27378798 PMC4972939

[B5] Bland M. (2015). *An Introduction to Medical Statistics*, 4th Edn. Oxford: Oxford University Press.

[B6] Cao W. Fang Z. Hou G. Han M. Xu X. Dong J. (2020). The psychological impact of the COVID-19 epidemic on college students in China. *Psychiatry Res.* 287:112934. 10.1016/j.psychres.2020.112934 32229390 PMC7102633

[B7] Chi X. Becker B. Yu Q. Willeit P. Jiao C. Huang L. (2020). Prevalence and psychosocial correlates of mental health outcomes among Chinese college students during the coronavirus disease (COVID-19) pandemic. *Front. Psychiatry* 11:803. 10.3389/fpsyt.2020.00803 32848958 PMC7427603

[B8] Christiansen D. M. (2015). “Examining sex and gender differences in anxiety disorders,” in *A Fresh Look at Anxiety Disorders*, ed. Durbano F. (Rijeka: InTech).

[B9] Dalgard O. S. (1996). “Community health profile as tool for psychiatric prevention,” in *Promotion of Mental Health*, Vol. 5 eds Trent D. R. Reed C. (Aldershot: Avebury).

[B10] Danneel S. Geukens F. Maes M. Bastin M. Bijttebier P. Colpin H. (2020). Loneliness, social anxiety symptoms, and depressive symptoms in adolescence: longitudinal distinctiveness and correlated change. *J. Youth Adolesc.* 49 2246–2264. 10.1007/s10964-020-01315-w 32918664

[B11] Dar K. A. Iqbal N. Mushtaq A. (2017). Intolerance of uncertainty, depression, and anxiety: examining the indirect and moderating effects of worry. *Asian J. Psychiatr.* 29 129–133. 10.1016/j.ajp.2017.04.017 29061409

[B12] Ding Y. Xu J. Huang S. Li P. Lu C. Xie S. (2020). Risk perception and depression in public health crises: evidence from the COVID-19 crisis in China. *Int. J. Environ. Res. Public Health* 17:5728. 10.3390/ijerph17165728 32784792 PMC7460398

[B13] Dratva J. Zysset A. Schlatter N. von Wyl A. Huber M. Volken T. (2020). Swiss university students’ risk perception and general anxiety during the COVID-19 pandemic. *Int. J. Environ. Res. Public Health* 17:7433. 10.3390/ijerph17207433 33066008 PMC7599649

[B14] Elmer T. Mepham K. Stadtfeld C. (2020). Students under lockdown: comparisons of students’ social networks and mental health before and during the COVID-19 crisis in Switzerland. *PLoS One* 15:e0236337. 10.1371/journal.pone.0236337 32702065 PMC7377438

[B15] Essadek A. Rabeyron T. (2020). Mental health of French students during the Covid-19 pandemic. *J. Affect. Disord.* 277 392–393. 10.1016/j.jad.2020.08.042 32861840 PMC7445153

[B16] Fancourt D. Steptoe A. Bu F. (2020). Trajectories of depression and anxiety during enforced isolation due to COVID-19: longitudinal analyses of 59,318 adults in the UK with and without diagnosed mental illness. *medRxiv* [Preprint]. 10.1101/2020.06.03.20120923

[B17] Federal Council (2020). *Bundesrat Verschärft Massnahmen gegen das Coronavirus zum Schutz der Gesundheit und unterstützt betroffene Branchen.* Available online at: https://www.admin.ch/gov/de/start/dokumentation/medienmitteilungen.msg-id-78437.html (accessed 31 July 2020).

[B18] Fichter M. M. Quadflieg N. Fischer U. C. Kohlboeck G. (2010). Twenty-five-year course and outcome in anxiety and depression in the Upper Bavarian Longitudinal Community Study. *Acta Psychiatr. Scand.* 122 75–85. 10.1111/j.1600-0447.2009.01512.x 19922523

[B19] Fullana M. A. Tortella-Feliu M. Fernández de la Cruz L. Chamorro J. Perez-Vigil A. Ioannidis J. P. A. (2020). Risk and protective factors for anxiety and obsessive-compulsive disorders: an umbrella review of systematic reviews and meta-analyses. *Psychol. Med.* 50 1300–1315. 10.1017/S0033291719001247 31172897

[B20] Giebel C. Corcoran R. Goodall M. Campbell N. Gabbay M. Daras K. (2020). Do people living in disadvantaged circumstances receive different mental health treatments than those from less disadvantaged backgrounds? *BMC Public Health* 20:651. 10.1186/s12889-020-08820-4 32393305 PMC7216680

[B21] Hamm A. O. (2019). Fear, anxiety, and their disorders from the perspective of psychophysiology. *Psychophysiology* 57:e13474. 10.1111/psyp.13474 31529522

[B22] Heinrichs N. Rapee R. M. Alden L. A. Bogels S. Hofmann S. G. Oh K. J. (2006). Cultural differences in perceived social norms and social anxiety. *Behav. Res. Ther.* 44 1187–1197. 10.1016/j.brat.2005.09.006 16325145

[B23] Hettema J. M. Prescott C. A. Myers J. M. Neale M. C. Kendler K. S. (2005). The structure of genetic and environmental risk factors for anxiety disorders in men and women. *Arch. Gen. Psychiatry* 62 182–189. 10.1001/archpsyc.62.2.182 15699295

[B24] Hibell B. Guttormsson U. Ahlström S. Balakireva O. Bjarnason T. Kokkevi A. (2009). *The 2007 ESPAD Report. Substance Use among Students in 35 European Countries.* Stockholm: European School Survey Project on Alcohol and Other Drugs.

[B25] Huang Y. Wang Y. U. Wang H. Liu Z. Yu X. Yan J. (2019). Prevalence of mental disorders in China: a cross-sectional epidemiological study. *Lancet Psychiatry* 6 211–224. 10.1016/S2215-0366(19)30128-2 30792114

[B26] Jeong H. Yim H. W. Song Y. J. Ki M. Min J. A. Cho J. (2016). Mental health status of people isolated due to Middle East Respiratory Syndrome. *Epidemiol. Health* 38:e2016048. 10.4178/epih.e2016048 28196409 PMC5177805

[B27] Jones J. H. Salathé M. (2009). Early assessment of anxiety and behavioral response to novel swine-origin influenza A(H1N1). *PLoS One* 4:e8032. 10.1371/journal.pone.0008032 19997505 PMC2779851

[B28] Kaparounaki C. K. Patsali M. E. Mousa D. P. V. Papadopoulou E. V. K. Papadopoulou K. K. K. Fountoulakis K. N. (2020). University students’ mental health amidst the COVID-19 quarantine in Greece. *Psychiatry Res.* 290:113111. 10.1016/j.psychres.2020.113111 32450416 PMC7236729

[B29] Kasteenpohja T. Marttunen M. Aalto-Setälä T. Perälä J. Saarni S. I. Suvisaari J. (2018). Outcome of depressive and anxiety disorders among young adults: results from the Longitudinal Finnish Health 2011 Study. *Nord. J. Psychiatry* 72 205–213. 10.1080/08039488.2017.1418429 29276896

[B30] Kirmayer L. J. Young A. Hayton B. C. (1995). The cultural context of anxiety disorders. *Psychiatr. Clin. North Am.* 18 503–521. 10.1016/S0193-953X(18)30037-6 8545264

[B31] Kocalevent R. D. Berg L. Beutel M. E. Hinz A. Zenger M. Harter M. (2018). Social support in the general population: standardization of the Oslo social support scale (OSSS-3). *BMC Psychol.* 6:31. 10.1186/s40359-018-0249-9 30016997 PMC6050647

[B32] Kontis V. Bennett J. E. Rashid T. Parks R. M. Pearson-Stuttard J. Guillot M. (2020). Magnitude, demographics and dynamics of the effect of the first wave of the COVID-19 pandemic on all-cause mortality in 21 industrialized countries. *Nat. Med.* 26 1919–1928. 10.1038/s41591-020-1112-0 33057181 PMC7615092

[B33] Lauriola M. Carleton R. N. Tempesta D. Calanna P. Socci V. Mosca O. (2019). A correlational analysis of the relationships among intolerance of uncertainty, anxiety sensitivity, subjective sleep quality, and insomnia symptoms. *Int. J. Environ. Res. Public Health* 16:3253. 10.3390/ijerph16183253 31491841 PMC6765836

[B34] Leung G. M. Ho L. M. Chan S. K. K. Ho S. Y. Bacon-Shone J. Choy R. Y. L. (2005). Longitudinal assessment of community psychobehavioral responses during and after the 2003 outbreak of severe acute respiratory syndrome in Hong Kong. *Clin. Infect. Dis.* 40 1713–1720. 10.1086/429923 15909256

[B35] Li H. Y. Cao H. Leung D. Y. P. Mak Y. W. (2020). The psychological impacts of a COVID-19 outbreak on college students in China: a longitudinal study. *Int. J. Environ. Res. Public Health* 17:3933. 10.3390/ijerph17113933 32498267 PMC7312488

[B36] Liu C. H. Zhang E. Wong G. T. F. Hyun S. Hahm H. C. (2020). Factors associated with depression, anxiety, and PTSD symptomatology during the COVID-19 pandemic: clinical implications for U.S. *young adult mental health*. *Psychiatry Res.* 290:113172. 10.1016/j.psychres.2020.113172 32512357 PMC7263263

[B37] Maaravi Y. Heller B. (2020). Not all worries were created equal: the case of COVID-19 anxiety. *Public Health* 185 243–245. 10.1016/j.puhe.2020.06.032 32688099 PMC7306738

[B38] McIntyre J. C. Worsley J. Corcoran R. Woods P. H. Bentall R. P. (2018). Academic and non-academic predictors of student psychological distress: the role of social identity and loneliness. *J. Ment. Health* 27 230–239. 10.1080/09638237.2018.1437608 29436883

[B39] McLaughlin K. A. Conron K. J. Koenen K. C. Gilman S. E. (2010). Childhood adversity, adult stressful life events, and risk of past-year psychiatric disorder: a test of the stress sensitization hypothesis in a population-based sample of adults. *Psychol. Med.* 40 1647–1658. 10.1017/S0033291709992121 20018126 PMC2891275

[B40] Naser A. Y. Dahmash E. Z. Al-Rousan R. Alwafi H. Alrawashdeh H. M. Ghoul I. (2020). Mental health status of the general population, healthcare professionals, and university students during 2019 coronavirus disease outbreak in Jordan: a cross−sectional study. *Brain Behav.* 10:e01730. 10.1002/brb3.1730 32578943 PMC7361060

[B41] Perz C. A. Lang B. A. Harrington R. (2020). Validation of the Fear of COVID-19 Scale in a US college Sample. *Int. J. Ment. Health Addict.* 10.1007/s11469-020-00356-3 [Epub ahead of print]. 32837435 PMC7315905

[B42] Price J. S. (2003). Evolutionary aspects of anxiety disorders. *Dialogues Clin. Neurosci.* 5 223–236. 22033473 10.31887/DCNS.2003.5.3/jprice PMC3181631

[B43] Raskind I. G. Haardörfer R. Berg C. J. (2019). Food insecurity, psychosocial health and academic performance among college and university students in Georgia, USA. *Public Health Nutr.* 22 476–485. 10.1017/S1368980018003439 30724722 PMC6366643

[B44] Savitsky B. Findling Y. Ereli A. Hendel T. (2020). Anxiety and coping strategies among nursing students during the Covid-19 pandemic. *Nurse Educ. Pract.* 46:102809. 10.1016/j.nepr.2020.102809 32679465 PMC7264940

[B45] Schneider R. L. Long E. E. Arch J. J. Hankin B. L. (2020). The relationship between stressful events, emotion dysregulation, and anxiety symptoms among youth: longitudinal support for stress causation but not stress generation. *Anxiety Stress Coping* 6 1–16. 10.1080/10615806.2020.1839730 33156724 PMC7904649

[B46] Sinclair V. G. Wallston K. A. (2004). The development and psychometric evaluation of the Brief Resilient Coping Scale. *Assessment* 11 94–101. 10.1177/1073191103258144 14994958

[B47] Sotomo. Corona-Krise: Monitoring der Bevölkerung 24/03/20 (2020). Available online at: https://sotomo.ch/site/corona-krise-monitoring-der-bevoelkerung/ (accessed October 12, 2020).

[B48] Spitzer R. L. Kroenke K. Williams J. B. W. Löwe B. (2006). A brief measure for assessing generalized anxiety disorder: the GAD-7. *Arch. Intern. Med.* 166:1092. 10.1001/archinte.166.10.1092 16717171

[B49] Stegmann Y. Schiele M. A. Schümann D. Lonsdorf T. B. Zwanzger P. Romanos M. (2019). Individual differences in human fear generalization-pattern identification and implications for anxiety disorders. *Transl. Psychiatry* 9 1–11. 10.1038/s41398-019-0646-8 31740663 PMC6861247

[B50] Tartas M. Walkiewicz M. Majkowicz M. Budzinski W. (2011). Psychological factors determining success in a medical career: a 10-year longitudinal study. *Med. Teach.* 33:e163-72. 10.3109/0142159X.2011.544795 21345056

[B51] Toufexis D. J. Myers K. M. Davis M. (2006). The effect of gonadal hormones and gender on anxiety and emotional learning. *Horm. Behav.* 50 539–549. 10.1016/j.yhbeh.2006.06.020 16904674

[B52] Wang C. Pan R. Wan X. Tan Y. Xu L. McIntyre R. S. (2020). A longitudinal study on the mental health of general population during the COVID-19 epidemic in China. *Brain Behav. Immun.* 87 40–48. 10.1016/j.bbi.2020.04.028 32298802 PMC7153528

[B53] Wang C. Zhao H. (2020). The impact of COVID-19 on anxiety in Chinese university students. *Front. Psychol.* 11:1168. 10.3389/fpsyg.2020.01168 32574244 PMC7259378

[B54] Wathelet M. Duhem S. Vaiva G. Baubet T. Habran E. Veerapa E. (2020). Factors associated with mental health disorders among university students in france confined during the COVID-19 pandemic. *JAMA Netw. Open* 3:e2025591. 10.1001/jamanetworkopen.2020.25591 33095252 PMC7584927

[B55] Zhang Y. Zhang H. Ma X. Di Q. (2020). Mental health problems during the COVID-19 pandemics and the mitigation effects of exercise: a longitudinal study of college students in China. *Int. J. Environ. Res. Public Health* 17:3722. 10.3390/ijerph17103722 32466163 PMC7277113

[B56] Zimmermann M. Bledsoe C. Papa A. (2020a). The impact of the COVID-19 pandemic on college student mental health: a longitudinal examination of risk and protective factors. *PsyArXiv* [Preprint]. 10.31234/osf.io/2y7hu PMC8556872 34763271

[B57] Zimmermann M. Chong A. K. Vechiu C. Papa A. (2020b). Modifiable risk and protective factors for anxiety disorders among adults: a systematic review. *Psychiatry Res.* 285:112705. 10.1016/j.psychres.2019.112705 31839417

